# A Novel Approach for Kalman Filter Tuning for Direct and Indirect Inertial Navigation System/Global Navigation Satellite System Integration

**DOI:** 10.3390/s24227331

**Published:** 2024-11-16

**Authors:** Adalberto J. A. Tavares Jr., Neusa M. F. Oliveira

**Affiliations:** Electronic Engineering Division, Aeronautics Institute of Technology, São José dos Campos 12228-900, Brazil; neusa@ita.br

**Keywords:** Kalman filter tuning, INS/GNSS integration, inertial sensor error model

## Abstract

This work presents an innovative approach for tuning the Kalman filter in INS/GNSS integration, combining states from the inertial navigation system (INS) and data from the Global Navigation Satellite System (GNSS) to enhance navigation accuracy. The INS uses measurements from accelerometers and gyroscopes, which are subject to uncertainties in scale factor, misalignment, non-orthogonality, and bias, as well as temporal, thermal, and vibration variations. The GNSS receiver faces challenges such as multipath, temporary signal loss, and susceptibility to high-frequency noise. The novel approach for Kalman filter tuning involves previously performing Monte Carlo simulations using ideal data from a predetermined trajectory, applying the inertial sensor error model. For the indirect filter, errors from inertial sensors are used, while, for the direct filter, navigation errors in position, velocity, and attitude are also considered to obtain the process noise covariance matrix Q. This methodology is tested and validated with real data from Castro Leite Consultoria’s commercial platforms, PINA-F and PINA-M. The results demonstrate the efficiency and consistency of the estimation technique, highlighting its applicability in real scenarios.

## 1. Introduction

The integration of an inertial navigation system (INS) and a Global Navigation Satellite System (GNSS) is an essential technique for delivering accurate position, velocity, and attitude estimates in both civil and military navigation applications. Effective state estimators are crucial to ensuring the accuracy of these estimates, with the Extended Kalman Filter (EKF) being one of the most widely adopted methods. The EKF can be used for nonlinear systems, where the model equations are linearized locally around the current state estimate during each iteration. The EKF assumes that both process noise and measurement noise are Gaussian, with zero mean [1]. Furthermore, it assumes that the covariance matrices of the process noise Q and the measurement noise R are known [2]. However, in real-world systems, these conditions are not always met, especially in INS/GNSS integration scenarios, leading to potential degradation in estimation accuracy.

The tuning of an EKF involves optimizing matrices Q and R to accurately estimate the states of a nonlinear system. This process starts with defining the initial state and covariance estimates, often based on system knowledge and initial measurements. The process noise covariance matrix Q and measurement noise covariance matrix R are crucial for the EKF performance and require careful tuning. These matrices are usually derived from empirical data or through iterative experimentation. Fine tuning involves adjusting Q and R to balance responsiveness to new measurements with stability against noisy data. Extensive simulation and field testing are typically required to ensure robustness across various operating conditions [3,4,5,6].

In INS/GNSS integration, the tuning process for an EKF is iterative and time-consuming. It usually begins with a static error estimate obtained through Allan variance analysis [7,8]. This initial estimate is often optimistic and requires extensive static data collection over many hours or even days, serving as a starting point for further adjustments. Subsequent tuning involves discrete parameter adjustments and evaluating the position drift during GNSS outages [7].

To establish the order of parameter tuning, the priority can be set by evaluating the drift caused by them during GNSS signal interruptions. These parameters need to be tested in various scenarios with variable dynamics, which is challenging without extensive field test data. Additionally, the size of the discrete steps chosen in their adjustments influences their final optimization. Consequently, the ideal parameters are rarely known, and those selected are often non-ideal [7,9,10].

Since the 1970s, various methods for estimating the covariance matrices Q and R have been proposed. Mehra [11,12] was a pioneer in this field, followed by Carew et al. [13] and Belanger [14]. Despite these initial advances, progress in tuning the covariance matrices stagnated for almost three decades. In 2006, Odelson et al. introduced the ALS (Autocovariance Least Squares) method for the estimation of Q and R, with subsequent improvements presented by Rajamani et al. [15] and Akesson et al. [16]. However, these methods presented limitations in Multiple-Input Multiple-Output (MIMO) systems, as demonstrated by Matisko [17]. In recent years, traditional methods have been refined, and new methods have been developed to improve the tuning of the Kalman filter. Allan variance, for example, has been used to characterize and model noise in inertial sensors, allowing for a better estimation of the Q matrix [18,19,20]. Additionally, neural networks have been employed to dynamically adjust the tuning parameters of the Kalman filter, adapting to changes in system characteristics and noise in real time [21,22,23]. Genetic algorithms have also been applied to optimize the tuning of the Kalman filter, exploring an ample space of possible values for Q and R and selecting the best parameters based on performance criteria [3,24,25].

This paper presents a novel approach for tuning the EKF, utilizing Monte Carlo simulations and taking advantage of the Central Limit Theorem (CLT). The Monte Carlo method enables the simulation and statistical analysis of filter tuning parameters across a diverse range of scenarios [26,27], providing a robust way to estimate uncertainties and complex system behaviors. The CLT ensures that, as the number of samples increases, the distribution of parameter estimates converges to a normal distribution [28,29], offering a theoretical basis for tuning the process noise covariance matrix Q of the EKF.

This work proposes using Monte Carlo simulations to calculate the covariance matrix Q, utilizing ideal data from inertial sensors along a trajectory in conjunction with their error models applied to navigation. This method allows for extracting the variances of the Q matrix for both direct and indirect filter implementations. This approach facilitates quicker and more precise filter tuning, resulting in more reliable position and velocity estimates, especially in scenarios where the GNSS signal is degraded. The results are compared with the traditional EKF tuning method and those obtained from the inertial platforms of Castro Leite Consultoria, PINA-F, and PINA-M.

The remainder of this paper is organized as follows: Section 2 explains the concepts of INS/GNSS integration. Section 3 details the novel approach for Kalman filter tuning methodology. Section 4 describes the testing procedure. Section 5 presents the simulations and experimental tests. Section 6 discusses the results obtained. Finally, Section 7 provides the concluding remarks.

## 2. INS/GNSS Concepts

This section will discuss the main concepts of INS/GNSS integration.

### 2.1. Inertial Navigation System (INS)

Inertial navigation is an autonomous process of calculating position by the double integration of acceleration at a point whose position needs to be determined [30]. This involves finding position, attitude, and velocity relative to the Earth or the inertial space.

An inertial navigation system (INS) typically consists of three accelerometers and three gyroscopes to measure accelerations in three dimensions and rotation rates around three axes. The development of Micro-Electro-Mechanical System (MEMS) technology has significantly expanded the application area of INS. Today, an Inertial Measurement Unit (IMU) generally includes a three-degree-of-freedom (3-DoF) gyroscope and a three-degree-of-freedom accelerometer, allowing for the precise measurement of motion and orientation in compact and cost-effective packages [31].

Nowadays, strapdown systems are more commonly used, where the gyroscopes and accelerometers are mounted directly to the structure of the vehicle or strapped onto the body segment. Strapdown mechanization (or INS mechanization) is the process of determining the navigation states (position, velocity, and attitude) from raw inertial measurements by solving the differential equations that describe the system’s motion. For this purpose, it is essential to establish a consistent reference frame.

The mechanization differential equations are formulated in the navigation frame, also known as the local-level frame or NED (North, East, Down) frame. This frame is fixed to the Earth’s surface, with its axes aligned to point towards geographic north, east, and downward (towards the center of the Earth). The ECEF (Earth-Centered Earth-Fixed) frame, in contrast, is a Cartesian coordinate system that rotates with the Earth, making it suitable for representing global positions in a geocentric reference system. The body frame is fixed to the vehicle itself, with its axes aligned along the forward, right, and downward directions of the vehicle. Finally, the inertial frame, specifically the Earth-Centered Inertial (ECI) frame, is centered at the Earth’s center and does not rotate with the Earth [32]. This work uses the WGS-84 model for the ECEF frame and the J2000 frame for the inertial reference.

The dynamic equations used for mechanization in the navigation frame are presented below and are detailed in [32]. In this context, the superscript indicates the frame in which the variable is expressed, and the subscript denotes the reference frame for the transformation: *n* refers to the navigation frame, *e* refers to the ECEF frame, *i* refers to the ECI frame, and *b* refers to the body frame.

1. Position Update:
  (1) $${\dot{p}}^{n}=D^{-1}v^{n}$$
  where $p^{n}={\left[\phi ,\lambda ,h\right]}^{T}$, with ϕ representing the latitude, λ the longitude, and *h* the height relative to the Earth ellipsoid. The matrix $D^{-1}$ is a 3×3 matrix that transforms the velocity vector from navigation coordinates to geodetic coordinates in the ECEF frame. The vector $v^{n}={\left[v_{n},v_{e},v_{d}\right]}^{T}$ represents the velocity in the NED frame, with components along the north ($v_{n}$), east ($v_{e}$), and down ($v_{d}$) directions.
2. Velocity Update:
  (2) $${\dot{v}}^{n}=R_{b}^{n}f^{b}+g^{n}-\left(\Omega_{en}^{n}+2\Omega_{ie}^{n}\right)v^{n}$$
  where $R_{b}^{n}$ is the direction cosine matrix from the body frame to the navigation frame, $f^{b}$ is the specific force measured by accelerometers in the body frame, $g^{n}$ is the gravity vector, $\Omega_{en}^{n}$ is the skew-symmetric matrix representing the transport rate with respect to the ECEF frame expressed in the navigation frame, and $\Omega_{ie}^{n}$ is the skew-symmetric matrix representing the Earth’s rotation rate with respect to the inertial frame expressed in the navigation frame.
3. Attitude Update:
  (3) $${\dot{R}}_{b}^{n}=R_{b}^{n}\left(\Omega_{ib}^{b}-\Omega_{in}^{b}\right)$$
  where $\Omega_{ib}^{b}$ is the skew-symmetric matrix representing the angular rate of the body frame with respect to the inertial frame expressed in the body frame, and $\Omega_{in}^{b}$ is the skew-symmetric matrix representing the angular rate of the navigation frame with respect to the inertial frame expressed in the body frame.

These equations allow the continuous update of navigation states based on the inertial sensor measurements. Strapdown systems offer advantages in terms of simplicity and reliability, as they avoid the complexities and potential failure points associated with gimbaled systems, which use mechanical gyroscopes and rotating platforms to stabilize and orient sensors.

For a comprehensive treatment of strapdown inertial navigation systems and the associated mechanization equations, refer to Savage [30], Farrell [10], and Groves [1].

### 2.2. Global Navigation Satellite System (GNSS)

The Global Navigation Satellite System (GNSS) is a technology that allows for the determination of position, velocity, and time anywhere on Earth using satellite signals. GNSS includes various satellite constellations, such as the United States’ GPS, Russia’s GLONASS, the European Union’s Galileo, and China’s BeiDou [33,34]. These systems work by transmitting radio signals from satellites in orbit around the Earth. GNSS receivers on the Earth’s surface capture these signals and use the differences in arrival times to calculate the receiver’s position. GNSS is widely used in applications such as terrestrial, aerial, and maritime navigation, precision agriculture, surveying, and environmental monitoring [35]. Modern GNSS receivers can utilize multiple constellations simultaneously, enhancing the accuracy and robustness of measurements.

Despite these advancements, GNSS is still subject to several types of errors that can affect measurement accuracy. The main errors include satellite ephemeris errors (inaccuracies in the transmitted orbital information), satellite clock errors, ionospheric delays (caused by the dispersion of GNSS signals in the ionosphere), tropospheric delays (due to the refraction of signals in the troposphere), multipath effects (signals reflected off surfaces before reaching the receiver), and receiver measurement errors [34].

### 2.3. INS/GNSS Integration

In INS/GNSS integration, the nominal trajectory is often unknown in advance. Therefore, the current best estimate of the actual trajectory is used as the nominal trajectory. When Kalman filtering is applied to a system that has been linearized around this estimate, it is known as the EKF [10,32,36]. INS/GNSS integration can be performed in two modes: direct (state model) and indirect (error state model). In direct integration, the Kalman filter directly estimates states such as position, velocity, and attitude. In indirect integration, the Kalman filter is used to estimate the errors of the state vector of the inertial navigation algorithm, and these errors are subsequently corrected. Additionally, two types of error feedback mechanisms are employed: open-loop and closed-loop. In the open-loop configuration, corrections to position, velocity, and attitude are applied externally to the INS, with the estimated errors subtracted from the INS solution at each iteration. In contrast, the closed-loop configuration feeds the EKF error estimates back into the system, continuously correcting the INS solution. This feedback keeps the INS errors small, ensuring that the linearity assumption required for the EKF technique is maintained throughout the process [32].

Furthermore, there are different levels of coupling in INS/GNSS integration. Loosely coupled integration processes the GNSS and INS data separately before combining them. This method is simple and efficient, but less robust in environments with degraded GNSS signals. Tightly coupled integration directly incorporates GNSS measurements into the INS navigation equations, providing greater accuracy and robustness when dealing with weak or intermittent GNSS signals [37]. Ultra-tightly coupled integration goes further, directly integrating raw GNSS signal measurements into the navigation filter, resulting in greater resilience to degraded GNSS environments and improved performance in high-dynamic scenarios [38]. The integration of INS and GNSS in this work is based on a loosely coupled approach with a closed-loop configuration, as the trajectory is not known in advance.

### 2.4. Extended Kalman Filter (EKF)

The Extended Kalman Filter (EKF) is a widely used algorithm for state estimation in nonlinear systems. It extends the traditional Kalman filter (KF), which is designed for linear systems, by linearizing around the current estimate to handle the nonlinearity. The following subsections presents the EKF equations for both direct and indirect navigation modes.

#### 2.4.1. Direct Mode (State Model)

In the Direct EKF (DEKF), also known as the state model, the states are estimated based on the mathematical model of the system rather than the error dynamics of the states [39]. Thus, the DEKF directly estimates the navigation states, which in this work are represented by the following state vector:

(4) $$x={\left[p^{T},\;v^{T},\;\epsilon^{T}\right]}^{T},$$

where ϵ represents the attitude in Euler angles, specifically roll (ϕ), pitch (θ), and yaw (ψ).

The general system of differential equations that governs the state-space model used by the EKF can be expressed as follows:

(5) $$\dot{x}\left(t\right)=f\left(x\left(t\right),u\left(t\right),t\right)+w\left(t\right),$$

(6) z(t)=h(x(t),t)+v(t),

where x(t) is the state vector as a function of the time *t*, u(t) is the deterministic forcing function, w(t) is the model noise, z(t) is the measurement vector, v(t) is the measurement noise, and f and h are nonlinear known functions [40].

The discrete state of process and measurements of the DEKF are defined as follows:

(7) $$x_{k}=\Phi_{k-1}x_{k-1}+w_{k-1},$$

(8) $$z_{k}=H_{k}x_{k}+v_{k},$$

where x represents the state vector as defined in Vector (4), Φ is the transition matrix, H is the observation matrix, and *k* denotes the discrete time step.

The transition matrix $\Phi_{k-1}$ can be found based on the first-order Taylor series approximation [41,42]:

(9) $$\Phi_{k-1}\approx I_{9}+\Delta t\left[\begin{array}{ccc} 0_{3\times 3} & I_{3} & 0_{3\times 3}\\ 0_{3\times 3} & 0_{3\times 3} & -F_{23}\\ 0_{3\times 3} & 0_{3\times 3} & F_{33} \end{array}\right],$$

$$F_{23}=R_{b}^{n}\cdot \left[\begin{array}{ccc} 0 & -f_{D} & f_{E}\\ f_{D} & 0 & -f_{N}\\ -f_{E} & f_{N} & 0 \end{array}\right],$$

$$F_{33}=\left[\begin{array}{ccc} 0 & -\omega_{D} & \omega_{E}\\ \omega_{D} & 0 & -\omega_{N}\\ -\omega_{E} & \omega_{N} & 0 \end{array}\right],$$

where

- Δt is the time step interval;
- $I_{9}$ is the 9 × 9 identity matrix;
- $I_{3}$ is the 3 × 3 identity matrix;
- $0_{3\times 3}$ is a 3 × 3 zero matrix;
- $R_{b}^{n}$ is the rotation matrix from body frame to navigation frame, which follows the 3-2-1 (yaw–pitch–roll) rotation sequence;
- $f_{N}$, $f_{E}$, and $f_{D}$ are components of specific force in the north, east, and down directions, respectively;
- $\omega_{N}$, $\omega_{E}$, and $\omega_{D}$ are the angular velocities in the north, east, and down directions, respectively.

In the next equations, $I_{m}$ represents an identity matrix of order *m*, and $0_{m\times n}$ represents a zero matrix of dimension m×n.

The observations of the algorithm consist of GNSS positions and velocities; therefore,

(10) $$H=\left[\begin{array}{ccc} I_{3} & 0_{3\times 3} & 0_{3\times 3}\\ 0_{3\times 3} & I_{3} & 0_{3\times 3} \end{array}\right].$$

Also, define the following:

(11) $$\begin{array}{cc} Q_{k-1} & =E[w_{k-1}w_{k-1}^{T}],\\ R_{k} & =E[v_{k}v_{k}^{T}],\\ P_{k-1}^{+} & =E[\left({\hat{x}}_{k-1}^{+}-x_{k-1}\right){\left({\hat{x}}_{k-1}^{+}-x_{k-1}\right)}^{T}],\\ P_{k}^{-} & =E[\left({\hat{x}}_{k}^{-}-x_{k}\right){\left({\hat{x}}_{k}^{-}-x_{k}\right)}^{T}],\\ P_{k}^{+} & =E[\left({\hat{x}}_{k}^{+}-x_{k}\right){\left({\hat{x}}_{k}^{+}-x_{k}\right)}^{T}], \end{array}$$

where E[·] is the expected value operator, and Q and R represent the covariance matrices of the process noise and measurement noise, respectively, P is error covariance matrix, the superscript (−) denotes the a priori estimate, and superscript (+) denotes the a posteriori estimate.

Once the process and measurement models are defined by the previous equations, the DEFK procedure can be summarized as follows:

1. Prediction—Estimates the states for the next time step using the accelerations and current angular velocity combined with the states produced by the DEKF for the previous time step:
  (12) $${\hat{x}}_{k}^{-}=\Phi_{k-1}{\hat{x}}_{k-1}^{+},$$
  (13) $$P_{k}^{-}=\Phi_{k-1}P_{k-1}^{+}\Phi_{k-1}^{T}+Q_{k-1}.$$
2. Kalman Gain—Finds the Kalman gain K based on the estimation produced in the Prediction step:
  (14) $$K_{k}=P_{k}^{-}H_{k}^{T}{\left(H_{k}P_{k}^{-}H_{k}^{T}+R_{k}\right)}^{-1}.$$
3. Update—Uses measurements at time step *k* and the Kalman gain to correct the estimation produced in the Prediction step:
  (15) $${\hat{x}}_{k}^{+}={\hat{x}}_{k}^{-}+K_{k}\left(z_{k}-H_{k}{\hat{x}}_{k}^{-}\right),$$
  (16) $$P_{k}^{+}=\left(I-K_{k}H_{k}\right)P_{k}^{-}.$$

#### 2.4.2. Indirect Mode (Error State Model)

In the Indirect EKF (IEKF), also known as the error state model, the EKF estimates the errors in the navigation states rather than the states themselves. This approach involves maintaining a reference trajectory and estimating deviations from this trajectory. Therefore, in this work, the navigation error states are represented by the error state vector:

(17) $$\delta x_{k}={\left[\delta p^{T},\;\delta v^{T},\;\delta \epsilon^{T}\right]}^{T}.$$

The continuous-time system dynamic model and measurement model of the IEKF are defined as follows:

(18) $$\delta \dot{x}\left(t\right)=f\left(\delta x\left(t\right),t\right)+Gw\left(t\right),$$

(19) z(t)=h(δx(t),t)+v(t),

where f and h are the nonlinear functions of the error state vector, G is the process noise gain matrix, and w and v represent process and measurement noise, respectively.

The discrete state of process and measurement models of the IEKF are defined as follows:

(20) $$\delta x_{k}=\Phi_{k-1}\delta x_{k-1}+G_{k-1}w_{k-1},$$

(21) $$z_{k}=H_{k}\delta x_{k}+v_{k},$$

where δx represents the error state vector as defined in Vector (17), z is the measurement vector, Φ is the transition matrix, H is the observation matrix, and and *k* denotes the discrete time step.

The transition matrix $\Phi_{k-1}$ can be found based on the first-order Taylor series approximation [10,42]:

(22) $$\Phi_{k-1}\approx I_{9}+\Delta t\left[\begin{array}{ccccccccc} 0 & 0 & \frac{\rho_{E}}{R_{e}} & \frac{1}{R_{e}} & 0 & 0 & 0 & 0 & 0\\ \frac{-\rho_{D}}{\cos(\phi )} & 0 & \frac{-\rho_{N}}{R_{e}\cos\left(\phi \right)} & 0 & \frac{1}{R_{e}\cos\left(\phi \right)} & 0 & 0 & 0 & 0\\ 0 & 0 & 0 & 0 & 0 & -1 & 0 & 0 & 0\\ F_{41} & 0 & F_{43} & k_{D} & 2\omega_{D} & -\rho_{E} & 0 & f_{D} & -f_{E}\\ F_{51} & 0 & F_{53} & F_{54} & F_{55} & F_{56} & -f_{D} & 0 & f_{N}\\ -2v_{E}\Omega_{D} & 0 & F_{63} & 2\rho_{E} & -2\omega_{N} & 0 & f_{E} & -f_{N} & 0\\ -\Omega_{D} & 0 & \frac{\rho_{N}}{R_{e}} & 0 & -\frac{1}{R_{e}} & 0 & 0 & \omega_{D} & -\omega_{E}\\ 0 & 0 & \frac{\rho_{E}}{R_{e}} & \frac{1}{R_{e}} & 0 & 0 & -\omega_{D} & 0 & \omega_{N}\\ F_{91} & 0 & \frac{\rho_{D}}{R_{e}} & 0 & \frac{\tan(\phi )}{R_{e}} & 0 & \omega_{E} & -\omega_{N} & 0 \end{array}\right],$$

$$\begin{array}{cccc} \Omega_{N} & =\omega_{ie}\cos\left(\phi \right) & F_{41} & =-2\Omega_{N}v_{e}-\frac{\rho_{N}v_{e}}{\cos^{2}\left(\phi \right)}\\ \Omega_{D} & =-\omega_{ie}\sin\left(\phi \right) & F_{43} & =\rho_{E}k_{D}-\rho_{N}\rho_{D}\\ \rho_{N} & =\frac{v_{e}}{R_{e}} & F_{51} & =2\left(\Omega_{N}v_{n}+\Omega_{D}v_{d}\right)+\frac{\rho_{N}v_{n}}{\cos^{2}\left(\phi \right)}\\ \rho_{E} & =-\frac{v_{n}}{R_{e}} & F_{53} & =-\rho_{E}\rho_{D}-k_{D}\rho_{N}\\ \rho_{D} & =-\frac{v_{e}\tan\left(\phi \right)}{R_{e}} & F_{54} & =-(\omega_{D}+\Omega_{D})\\ \omega_{N} & =\Omega_{N}+\rho_{N} & F_{55} & =k_{D}-\rho_{E}\tan\left(\phi \right)\\ \omega_{E} & =\rho_{E} & F_{56} & =\omega_{N}+\Omega_{N}\\ \omega_{D} & =\Omega_{D}+\rho_{D} & F_{63} & =\rho_{N}^{2}+\rho_{E}^{2}-\frac{2g}{R_{e}}\\ k_{D} & =\frac{v_{d}}{R_{e}} & F_{91} & =\Omega_{N}+\frac{\rho_{N}}{\cos{\left(\phi \right)}^{2}} \end{array}$$

where

- $R_{e}$ is the Earth’s mean radius;
- $\omega_{ie}$ is the Earth’s rotation rate;
- $\Omega_{N}$ and $\Omega_{D}$ are components of the angular velocity due to Earth’s rotation;
- $\rho_{N}$, $\rho_{E}$, and $\rho_{D}$ are components of the relative velocity with respect to the Earth’s surface;
- $v_{e}$, $v_{n}$, and $v_{d}$ are the eastward, northward, and downward components of the velocity, respectively;
- ϕ is the latitude;
- $\omega_{N}$, $\omega_{E}$, and $\omega_{D}$ are the angular velocities incorporating both the Earth’s rotation and the relative velocities;
- $f_{N}$, $f_{E}$, and $f_{D}$ are components of specific force;
- $k_{D}$ is a component of the velocity.

The matrix G projects the process noise w into the state space, accounting for uncertainty in the state estimation [10], thus:

(23) $$G=\left[\begin{array}{cc} 0_{3\times 3} & 0_{3\times 3}\\ -R_{b}^{n} & 0_{3\times 3}\\ 0_{3\times 3} & R_{b}^{n} \end{array}\right],$$

The observations of the algorithm consist of GNSS positions and velocities, using the same matrix as in Equation (10).

Also, define the following:

(24) $$\begin{array}{cc} Q_{k-1} & =E[w_{k-1}w_{k-1}^{T}],\\ R_{k} & =E[v_{k}v_{k}^{T}],\\ P_{k-1}^{+} & =E[\left(\delta {\hat{x}}_{k-1}^{+}-\delta x_{k-1}\right){\left(\delta {\hat{x}}_{k-1}^{+}-\delta x_{k-1}\right)}^{T}],\\ P_{k}^{-} & =E[\left(\delta {\hat{x}}_{k}^{-}-\delta x_{k}\right){\left(\delta {\hat{x}}_{k}^{-}-\delta x_{k}\right)}^{T}],\\ P_{k}^{+} & =E[\left(\delta {\hat{x}}_{k}^{+}-\delta x_{k}\right){\left(\delta {\hat{x}}_{k}^{+}-\delta x_{k}\right)}^{T}], \end{array}$$

Once the process and measurement models are defined by the previous equations, the IEFK procedure can be summarized as follows:

1. Prediction—Estimates the states for the next time step using the accelerations and current angular velocity combined with the states produced by the IEKF for the previous time step:
  (25) $$\delta {\hat{x}}_{k}^{-}=\Phi_{k-1}\delta {\hat{x}}_{k-1},$$
  (26) $$P_{k}^{-}=\Phi_{k-1}P_{k-1}^{+}\Phi_{k-1}^{T}+G_{k-1}Q_{k-1}G_{k-1}^{T}.$$
2. Kalman Gain—Finds the Kalman gain K based on the estimation produced in the Prediction step:
  (27) $$K_{k}=P_{k}^{-}H_{k}^{T}{\left(H_{k}P_{k}^{-}H_{k}^{T}+R_{k}\right)}^{-1}.$$
3. Update—Uses measurements at time step *k* and the Kalman gain to correct the estimation produced in the Prediction step:
  (28) $$\delta {\hat{x}}_{k}^{+}=\delta {\hat{x}}_{k}^{-}+K_{k}\left(\delta z_{k}-H_{k}\delta {\hat{x}}_{k}^{-}\right),$$
  (29) $$P_{k}^{+}=\left(I-K_{k}H_{k}\right)P_{k}^{-}.$$

where $\delta z_{k}$ represents the difference between the positions and velocities estimated by the INS and those measured by the GNSS.

### 2.5. Standard Tuning of INS/GNSS EKF

In this subsection, the discussion is based on the work presented by Groves [1].

The primary sources of system noise in the INS are the random walk of velocity error caused by noise in the accelerometer’s specific-force measurements and the random walk of attitude error due to noise in the gyroscope’s angular-rate measurements. Additionally, if separate dynamic bias states for the accelerometer and gyroscope are not estimated, the in-run variation in the accelerometer and gyroscope biases can be approximated as white noise.

For small propagation intervals (≤0.2 s), the system noise covariance matrix can be approximated using Equation (30) for the DEKF and Equation (31) for the IEKF, where $S_{rg}$ and $S_{ra}$ are the variances of the gyroscope random noise and accelerometer random noise, respectively.

(30) $$Q_{INS_{dekf}}=\left[\begin{array}{ccc} 0_{3\times 3} & 0_{3\times 3} & 0_{3\times 3}\\ 0_{3\times 3} & S_{ra}I_{3} & 0_{3\times 3}\\ 0_{3\times 3} & 0_{3\times 3} & S_{rg}I_{3} \end{array}\right]$$

(31) $$Q_{INS_{iekf}}=\left[\begin{array}{cc} S_{ra}I_{3} & 0_{3\times 3}\\ 0_{3\times 3} & S_{rg}I_{3} \end{array}\right]$$

The power spectral densities (PSDs) of accelerometers and gyroscopes at frequencies below 1 Hz are generally white, meaning the standard deviation of the average specific force and angular rate noise is inversely proportional to the square root of the averaging time. As a result, inertial sensor noise is typically characterized by the root of PSD. The common units used are μg/$\sqrt{\mathrm{Hz}}$ for accelerometer noise, where 1 μg/$\sqrt{\mathrm{Hz}}=9.80665\times 10^{-6}$ m s^−1.5^, and ${}^{\circ}/\sqrt{\mathrm{hr}}$ or ${}^{\circ}/\mathrm{hr}/\sqrt{\mathrm{Hz}}$ for gyroscope noise, with $1^{\circ }/\mathrm{hr}=2.909\times 10^{-4}$ rad s^−0.5^ and $1^{\circ }/\sqrt{\mathrm{Hz}}=4.848\times 10^{-6}$ rad s^−0.5^. The standard deviations of these random noise samples are obtained by multiplying the corresponding root PSDs by the square root of the sampling rate, or by dividing by the square root of the sampling interval. Since the variance is the square of the standard deviation, this process also allows for the determination of the noise variance over the sampling period. It is important to note that white random noise cannot be calibrated or compensated for, as it lacks correlation between past and future values.

The tuning of the matrix R will not be discussed in this work, as its values will be derived from the GNSS receiver data. Consequently, the novel approach proposed for tuning the INS/GNSS integration will focus on the matrix Q and will be discussed in the next section.

## 3. Novel Approach EKF Tuning

The proposed EKF tuning method for INS/GNSS integration in both direct and indirect modes is based on the Monte Carlo technique, using ideal data from a predetermined trajectory and applying the inertial sensor error model to it. The method presented in this paper involves generating multiple trajectory measurements from a reference trajectory, with each measurement incorporating different random values of the accelerometer and gyroscope error model coefficients. These simulations are used to pre-calculate the variance matrices Q for both EKF modes.

**Figure 1:**
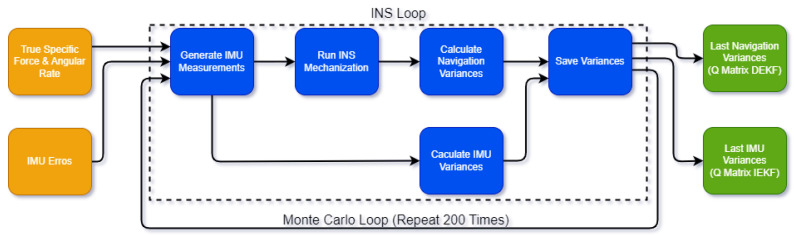
Flow diagram of the Monte Carlo simulation algorithm.

Figure 1 illustrates the flow of the Monte Carlo simulation process. The process begins with the use of the **true specific force and angular rate** from a reference trajectory, which are then combined with **IMU errors** to generate **IMU measurements**. Next, the **INS mechanization** is performed to estimate the navigation states. During the INS loop, the **navigation variances** and **IMU variances** are calculated and saved. The process is repeated 200 times, and the final variances produced yield the **navigation variances** for the DEKF and the **IMU variances** for the IEKF.

The color coding is as follows: orange blocks represent inputs, blue blocks represent the main computational steps of the Monte Carlo simulation, which include the INS loop, and green blocks represent the output variance matrices for both EKF modes.

### 3.1. Mathematical Modeling

In this subsection, the mathematical equations required for the implementation of the algorithm are discussed. Detailed explanations of the methods and equations used to ensure its proper functioning are provided.

For the input data, ideal inertial sensor data are used, which can be obtained through the trajectory generators proposed by Savage [43] or tools like MATLAB’s Navigation Toolbox. The IMU errors are derived from the manufacturer’s datasheet, and the provided data are implemented accordingly. The next topic will discuss the IMU errors in detail and explain how they are integrated into the system.

#### 3.1.1. Inertial Sensor Error Characteristics

In this subsection, the following equations are based on the work presented by Groves [1].

The accelerometer error model is given by Equation (32), which accounts for bias ($b_{a}$), errors due to the scale factor and cross-coupling ($M_{a}$, as shown in Equation (35)), and random noise ($w_{a}$):

(32) $${\tilde{f}}_{ib}^{b}=b_{a}+\left(I_{3\times 3}+M_{a}\right)f_{ib}^{b}+w_{a},$$

where $f_{ib}^{b}$ is the ideal specific force expressed in the body frame, obtained from the trajectory, and ${\tilde{f}}_{ib}^{b}$ is the computed specific force from the IMU.

The gyroscope error model is given by Equation (33), where the considered stochastic variables are bias ($b_{g}$), errors due to the scale factor and cross-coupling ($M_{g}$, as shown in Equation (35)), g-dependent bias ($G_{g}$), and random noise ($w_{g}$):

(33) $${\tilde{\omega }}_{ib}^{b}=b_{g}+\left(I_{3\times 3}+M_{g}\right)\omega_{ib}^{b}+G_{g}f_{ib}^{b}+w_{g},$$

where $\omega_{ib}^{b}$ is the ideal angular rate expressed in the body frame, also obtained from the trajectory, and ${\tilde{\omega }}_{ib}^{b}$ is the computed angular rate vector from the IMU.

When considering biases, it can be divided into static components, $b_{as}$ and $b_{gs}$, and dynamic components, $b_{ad}$ and $b_{gd}$, where

(34) $$b_{a}=b_{as}+b_{ad}\;\mathrm{and}\;b_{g}=b_{gs}+b_{gd}.$$

The static component, also referred to as the fixed bias, turn-on bias, or bias repeatability, encompasses the run-to-run variation in each instrument bias along with the residual fixed bias that remains after sensor calibration. This component remains constant during the IMU’s operation but can differ between runs. The dynamic component, also termed in-run bias variation or bias instability, changes over periods in the order of a minute and includes the residual temperature-dependent bias left after sensor calibration. Typically, the dynamic bias constitutes about 10% of the static bias [1].

The scale factor and cross-coupling errors for a nominally orthogonal set of accelerometers and gyroscopes can be represented by the following matrices:

(35) $$M_{a}=\left(\begin{array}{ccc} s_{a,x} & m_{a,xy} & m_{a,xz}\\ m_{a,yx} & s_{a,y} & m_{a,yz}\\ m_{a,zx} & m_{a,zy} & s_{a,z} \end{array}\right),\;M_{g}=\left(\begin{array}{ccc} s_{g,x} & m_{g,xy} & m_{g,xz}\\ m_{g,yx} & s_{g,y} & m_{g,yz}\\ m_{g,zx} & m_{g,zy} & s_{g,z} \end{array}\right),$$

where the accelerometer and gyroscope scale factor errors within the IMU are represented by the vectors $s_{a}=\left(s_{a,x},s_{a,y},s_{a,z}\right)$ and $s_{g}=\left(s_{g,x},s_{g,y},s_{g,z}\right)$, respectively. Additionally, $m_{a,\alpha \beta }$ represents the cross-coupling coefficient for the specific force along the β-axis sensed by the α-axis accelerometer, and $m_{g,\alpha \beta }$ represents the cross-coupling coefficient for the angular rate along the β-axis sensed by the α-axis gyroscope. Scale factor and cross-coupling errors are dimensionless and are typically expressed in parts per million (ppm) or as a percentage.

Once the inertial sensor data are obtained, their variances are calculated incrementally and the data are used in the INS mechanization equations.

#### 3.1.2. INS Mechanization

For INS mechanization using Equations (1)–(3), the attitude is propagated using quaternions. These equations are a simplification of those presented by Savage [30], where they are detailed in both continuous and discrete forms. In this work, the continuous form of the equations was used, with the fourth-order Adams–Bashforth predictor method applied for numerical integration. A detailed explanation of these equations is beyond the scope of this work.

Propagation of attitude using quaternions is just one part of the INS process; understanding and accounting for gravitational effects is equally critical. Since accelerometers cannot distinguish the effects of the gravitational field in their acceleration measurements, accurate knowledge of the gravitational field is necessary for INS calculations. The gravity model used in this work is based on the WGS-84 standard, which accounts for the Earth’s oblateness. This model estimates gravitational components based on latitude, longitude, and height (lat, long, h) relative to the Earth’s center.

The results of the INS mechanization equations yield positions in terms of latitude, longitude, and height, with velocities expressed in the NED coordinate frame. Additionally, the variances of positions, velocities, and attitude are calculated incrementally. A detailed explanation of this incremental calculation will be provided in the next subsection.

#### 3.1.3. Monte Carlo Simulation

The algorithm implements Welford’s method [44] for the incremental estimation of the mean and variance of a dataset, incorporating Bessel’s correction [45] for samples. These techniques are useful when dealing with large sequential datasets, and when efficient and accurate calculations of means and variances are needed.

Welford’s mean is calculated incrementally, allowing the mean to be updated as new elements are added to the dataset. The formula is as follows:

(36) $${\bar{x}}_{n}={\bar{x}}_{n-1}+\frac{x_{n}-{\bar{x}}_{n-1}}{n},$$

where *n* is the number of elements processed so far, $x_{n}$ is the most recent element, and ${\bar{x}}_{n}$ is the mean up to that point.

Welford’s variance is a natural extension, including Bessel’s correction to compensate for bias in the variance estimation when dealing with samples. The formula is as follows:

(37) $$s_{n}^{2}=\frac{n-1}{n}\cdot s_{n-1}^{2}+\frac{1}{n}\cdot {\left(x_{n}-{\bar{x}}_{n-1}\right)}^{2},$$

where *n* is also the number of elements processed so far, $s_{n}^{2}$ is the variance up to that point, and $s_{n-1}^{2}$ is the variance from the previous step.

By applying all the above equations, which encompass the inertial sensor error model, the INS mechanization equations, and the calculations for means and variances, the algorithm for the novel approach to Kalman filter tuning is described as presented in Algorithm 1:
**Algorithm 1:** Monte Carlo Simulation
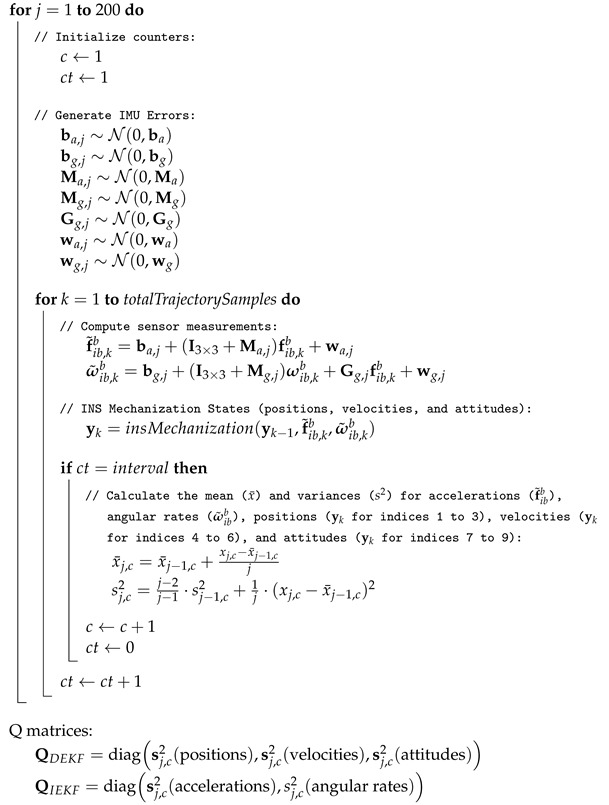

where *j* is the index of the Monte Carlo loop, *k* is the index for the trajectory samples, and *c* is the index used to calculate the means and variances for each trajectory step according to the chosen interval, which in this case is 1 s. Additionally, ct is an auxiliary index that counts the number of seconds until the specified interval is reached. *totalTrajectorySamples* represents the total number of samples in the trajectory dataset, *insMechanization* is a function that calculates the INS states based on the current states, and *diag* represents a diagonal matrix where the provided elements are placed along the main diagonal, with all off-diagonal elements set to zero.

The sensor error model is derived from the IMU datasheet, where not all error parameters need to be filled out. However, the more variables from the error equations that are defined, the more accurate the variance calculations will be. To introduce variability in the Monte Carlo simulation, the IMU errors are modeled as normally distributed random variables, each with a variance specific to its corresponding error parameter.

At the conclusion of the Monte Carlo simulation, the final variance values associated with the inertial sensors, accelerometers, and gyroscopes form the covariance matrix Q for the IEKF. Similarly, the final variance values related to navigation parameters, such as positions, velocities, and attitudes, form the covariance matrix Q for the DEKF.

Modeling inertial sensor errors is complex due to their non-Gaussian nature. In many applications, the Monte Carlo method generates samples assuming these errors follow a Gaussian distribution. In reality, inertial sensor errors often exhibit time-correlated characteristics, and a more accurate representation would involve using models such as a Markov process. To explain the accuracy and reliability of statistical analysis in Monte Carlo simulations for a non-Gaussian distribution, fundamental statistical principles are applied, one of which is the CLT.

### 3.2. Central Limit Theorem

The Central Limit Theorem (CLT) supports the proposed method by ensuring that, as the number of samples increases, the distribution of the sample tends to approach a normal distribution. This is particularly beneficial in Monte Carlo simulations, where the noise modeled by numerous random processes aggregates into a Gaussian distribution, simplifying the analysis and enhancing the reliability of statistical measures such as the covariance matrices derived from the simulation. This alignment of the sample distribution with the properties of Gaussian noise helps in making more accurate and robust predictions about the behavior of INS erros.

As discussed by Dobrushin [46] and Trevezas and Limnios [47], the application of the CLT to the Markov process is particularly relevant, as it allows for the modeling of complex stochastic processes where the transition probabilities change over time, ensuring that asymptotic normality can be achieved under certain conditions. This is especially pertinent for inertial sensor errors, which often follow a Markov process. These errors, including biases and random drifts in accelerometers and gyroscopes, can be modeled as first-order Markov processes [1,10,30]. This modeling captures the temporal correlations inherent in the sensor errors, allowing for more accurate tracking and prediction of navigation parameters.

In conclusion, the CLT within Monte Carlo simulations ensures that the distribution of tuning parameters estimates tends toward normality, even in complex stochastic processes like those modeled by first-order Markov processes. This approach simplifies the analysis and ensures that predictions regarding INS errors converge to values closer to the real ones, leading to more reliable INS/GNSS integrations.

## 4. Testing Procedure

**Figure 2:**
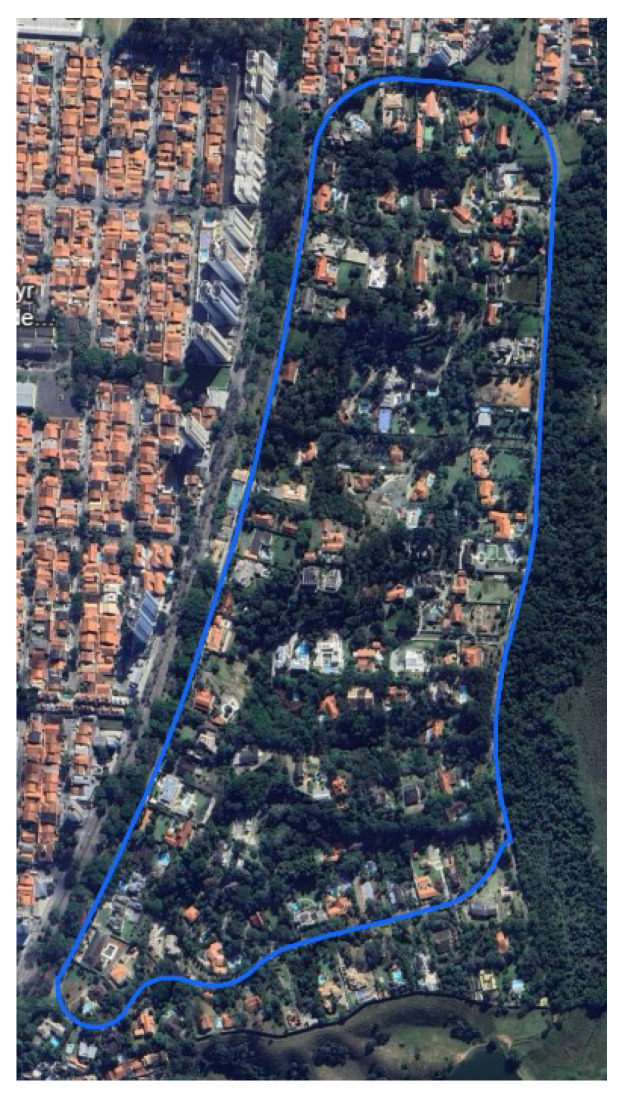
Top-down view of test trajectory highlighted in blue.

**Figure 3:**
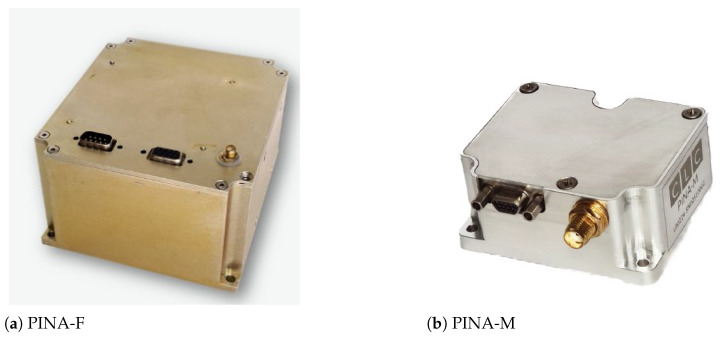
Castro Leite Consultoria’s inertial platforms.

The testing was conducted in a condominium located in São José dos Campos, Brazil, using a car. The test trajectory is illustrated in Figure 2. Two inertial navigation platforms with GPS assistance from Castro Leite Consultoria were used: the PINA-F and the PINA-M, as shown in Figure 3. The PINA-F, which is at Technology Readiness Level (TRL) 9, is equipped with Fiber Optic Gyroscopes (FOGs) and Quartz Pendulous Accelerometers (QPAs), while the PINA-M, at TRL 8, employs MEMS technology for its sensors (accelerometers and gyroscopes). The specifications for each platform can be seen in Table 1 and Table 2.

Four laps in the condominium were conducted for each platform, during which the GPS signal was intentionally suppressed at certain parts of the trajectory. The navigation results of the platforms are compared using algorithms with DEKF and IEKF under standard tuning conditions, as well as with those obtained using the novel tuning approach proposed in this study. These results will be discussed in the next section.

## 5. Results

This section presents the Q matrix values derived from the proposed novel approach for Kalman filter tuning in both direct and indirect INS/GNSS integration. Additionally, the other parameters used to initialize the EKFs are detailed, along with the results obtained from the filter tunings in both the standard (STD) form and the proposed new approach (NA). These results are then compared with those obtained from the platforms.

To calculate the variances in the Q matrix using the NA tuning method, inertial data from a roller coaster trajectory, employed in the SIA project (Inertial Systems for Aerospace Application, Sistemas Inerciais para Aplicação Aeroespacial in Portuguese) at the Brazilian Institute of Aeronautics and Space (IAE, Instituto de Aeronáutica e Espaço in Portuguese), are used as the reference trajectory in the Monte Carlo simulation. This trajectory induces more pronounced accelerations and angular velocities, leading to larger variances. This approach is specifically designed to tune the filter for worst-case scenarios, thereby ensuring robustness under extreme conditions. The variance results, truncated to the fourth decimal place, are presented in Table 3 and Table 4 for the DEKF and IEKF, respectively. For the DEKF variances, it can be observed that the position variance values were high for the vertical channel due to its instability, a phenomenon discussed in detail by Savage et al. [30] in the context of inertial navigation systems.

For the other EKF parameters, all filters used the same calibrated inertial sensor data from the platforms, the same value for the matrix R, and identical initial point values in the INS for each run, with all data originating from the platforms. For the initial values of the covariance matrix P, all filters used the same values, except for those compared with the PINA-F platform. This exception was necessary because the PINA-F platform does not provide standard deviation data in its log.

The following subsections present the results obtained from the filter tunings. The reference trajectory, shown in some graphs, was obtained using Google Earth and is therefore only applicable to latitude and longitude plots. In the graph legends, “STD Q” denotes the standard tuning, while “NA Q” represents the tuning based on the novel approach proposed in this work. To enhance visualization, areas where the GNSS signal experienced significant degradation are enlarged in the graphs.

### 5.1. DEKF

This subsection will present the results of the tunings for the direct filter.

#### 5.1.1. PINA-F

**Figure 4:**
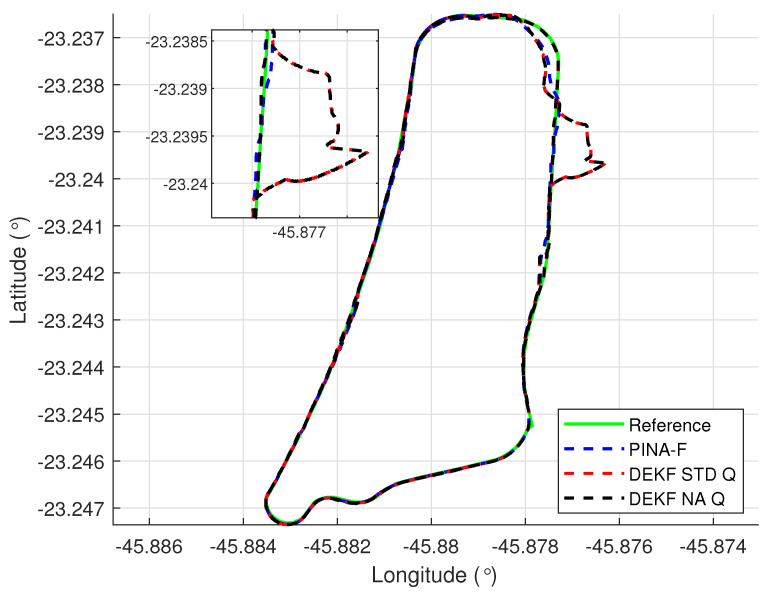
Geodetic position.

**Figure 5:**
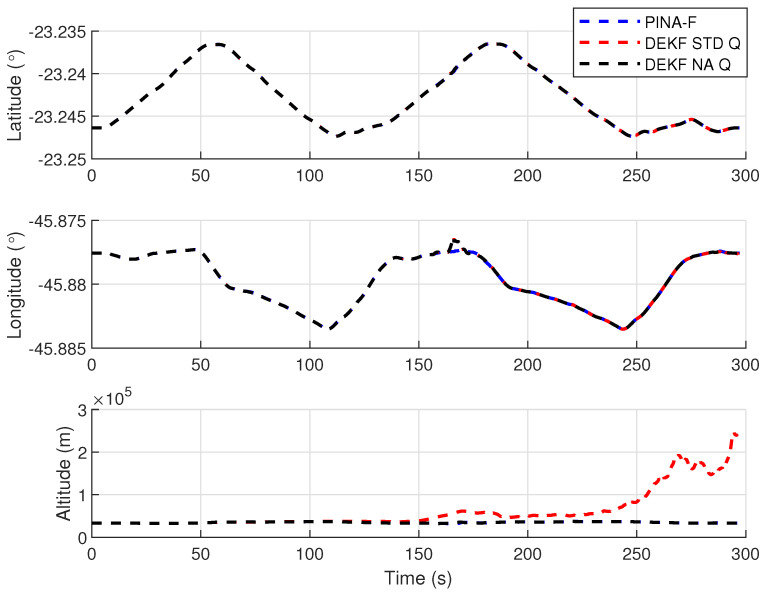
Positions in geodetic frame.

**Figure 6:**
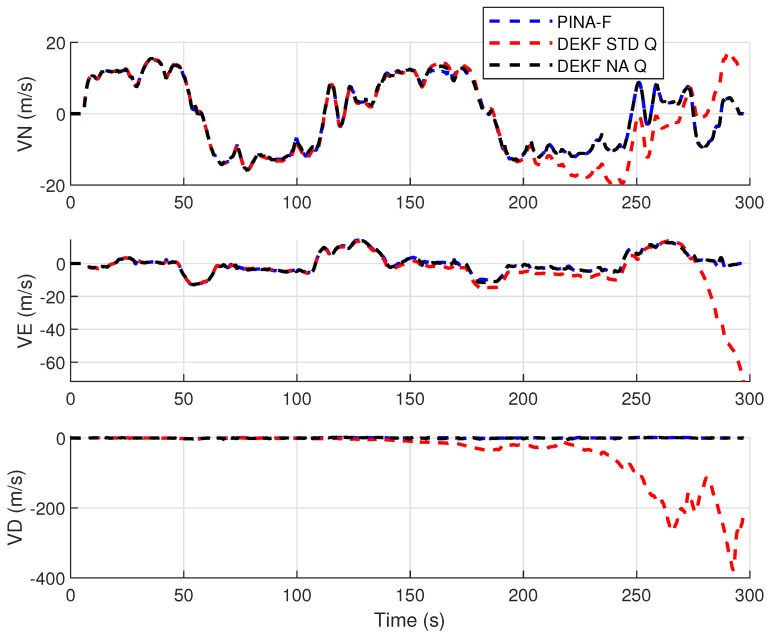
Velocities in navigation frame.

**Figure 7:**
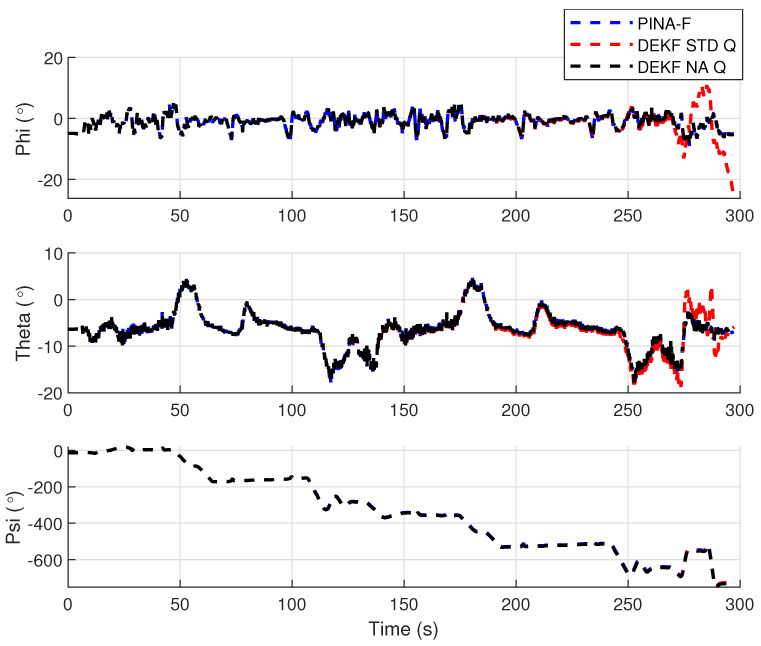
Euler angles.

Figure 4 presents the results of the INS/GNSS integration for the PINA-F in the geodetic reference frame. No significant differences are observed between the STD and NA tuning. However, Figure 5, Figure 6 and Figure 7 reveal variations when the axes are analyzed independently. In Figure 5, there is degradation in altitude with the STD tuning, while the NA tuning maintains closer alignment with the platform results. In Figure 6, the filter with the NA tuning follows the platform results, whereas the STD tuning shows discrepancies across all three axes. Regarding the Euler angles, presented in Figure 7, the NA tuning shows results close to those of the platform, while the STD tuning diverges at the end of the trajectory, particularly in the roll and pitch angles.

#### 5.1.2. PINA-M

**Figure 8:**
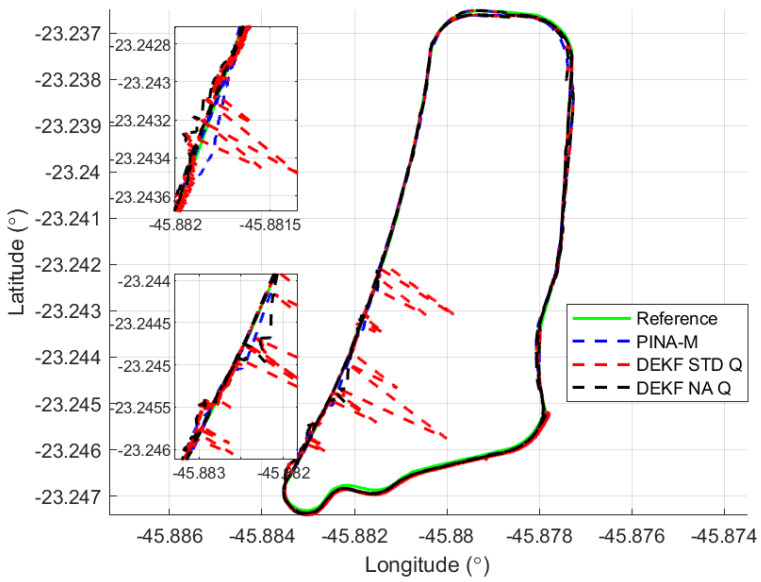
Geodetic position.

**Figure 9:**
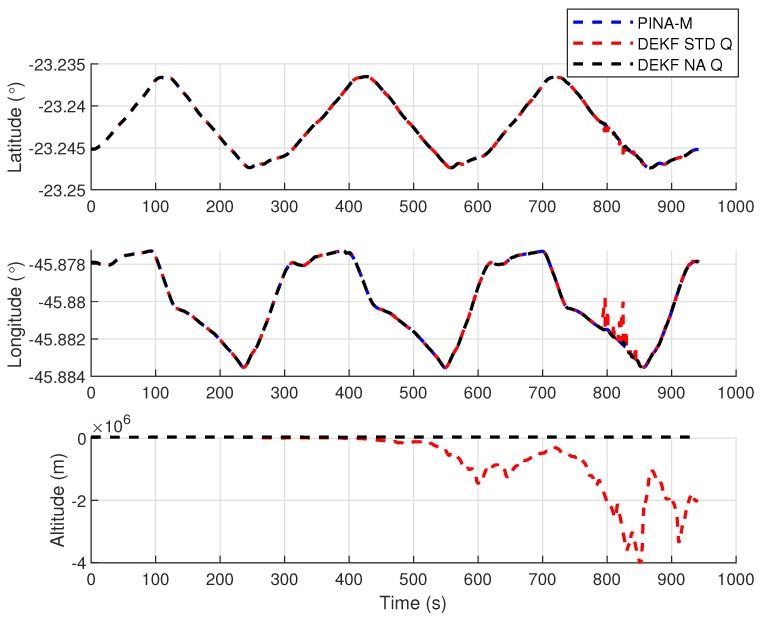
Positions in geodetic frame.

**Figure 10:**
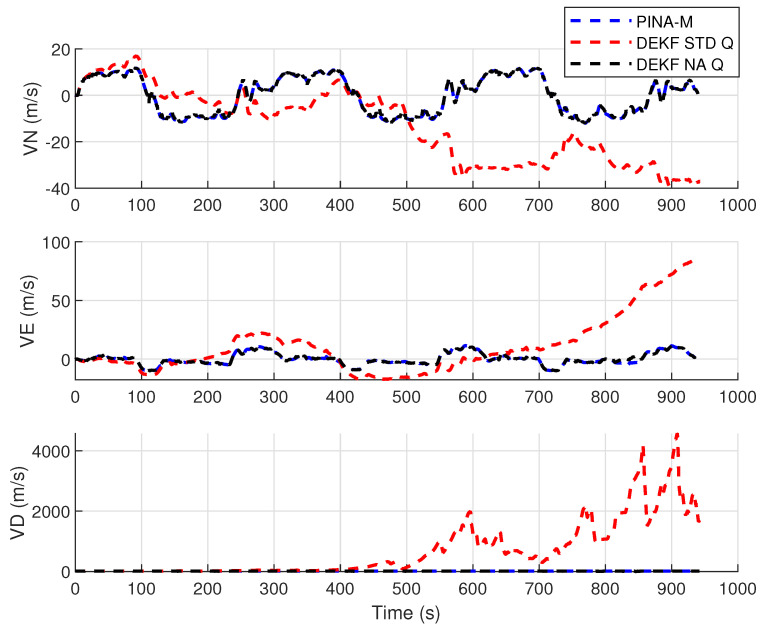
Velocities in navigation frame.

**Figure 11:**
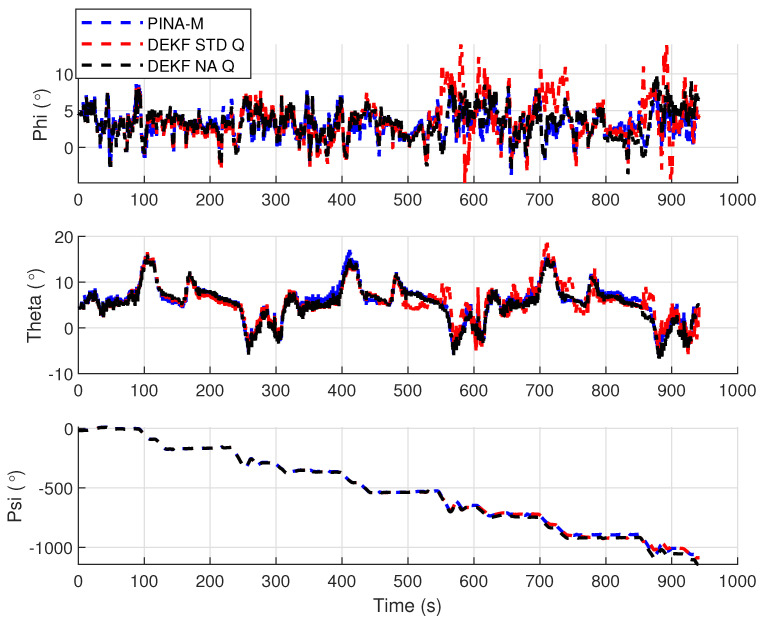
Euler angles.

Figure 8 presents the results of the INS/GNSS integration for the PINA-M in the geodetic reference frame. The NA tuning maintained the trajectory even during moments of GNSS signal interference, achieving better results in some parts of the trajectory than the platform itself, as seen in the enlarged area between latitudes −23.2428 and −23.2436 and longitudes −45.882 and −45.8815. In Figure 9, there is degradation in altitude with the STD tuning, and in latitude and longitude during periods without GNSS, while the NA tuning maintains closer alignment with the platform results. In Figure 10, the filter with the NA tuning follows the platform results, whereas the STD tuning shows discrepancies across all three axes. For the Euler angles, presented in Figure 11, the tunings show similar results overall, but the STD tuning exhibits greater divergence in the roll angle.

### 5.2. IEKF

This subsection will present the results of the tunings for the indirect filter.

#### 5.2.1. PINA-F

**Figure 12:**
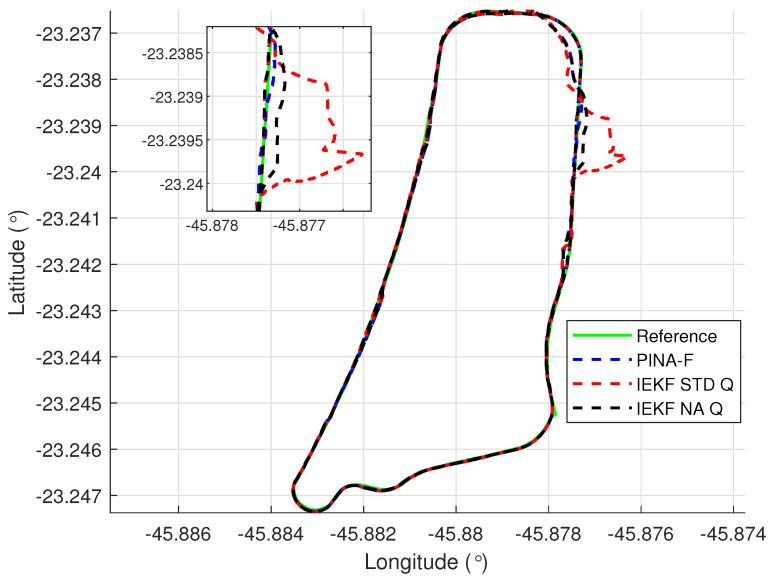
Geodetic position.

**Figure 13:**
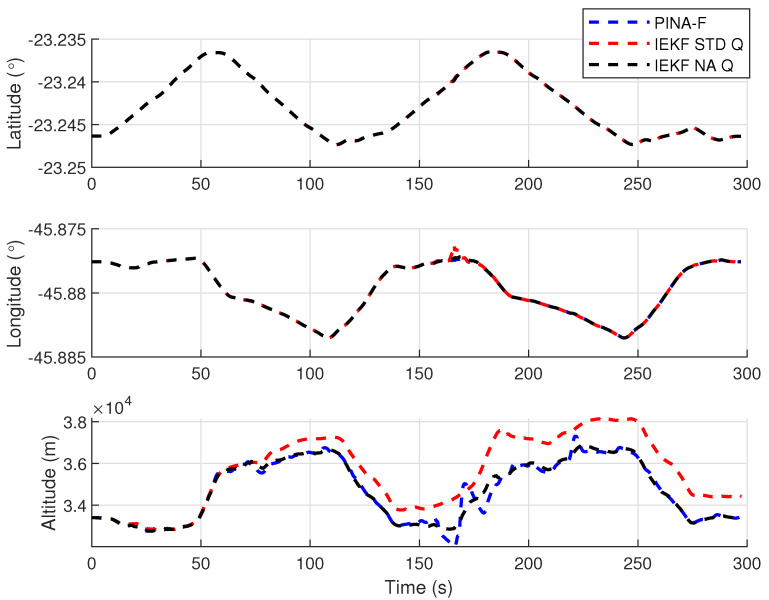
Positions in geodetic frame.

**Figure 14:**
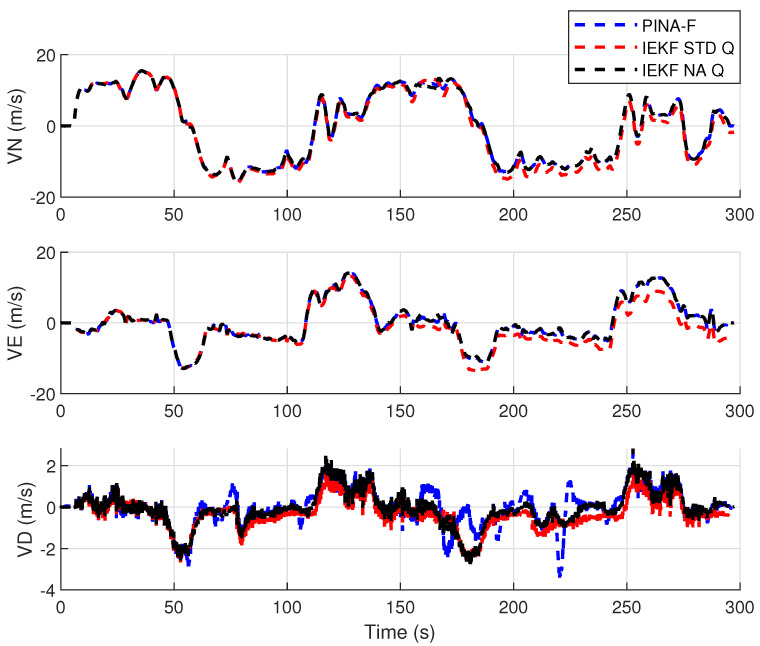
Velocities in navigation frame.

**Figure 15:**
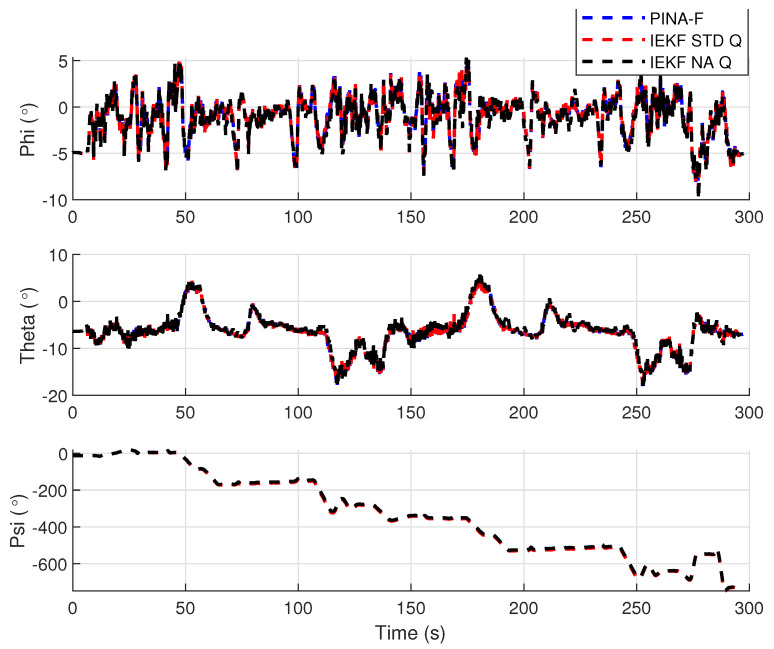
Euler angles.

Figure 12 presents the results of the INS/GNSS integration for the PINA-F in the geodetic reference frame. The NA tuning demonstrates a smaller error compared to the STD tuning, as shown in the enlarged area. In Figure 13, there is degradation in altitude with the STD tuning, while the NA tuning maintains closer alignment with the platform’s results. In Figure 14, the filter with the NA tuning follows the platform results for north and east velocities, whereas the STD tuning shows discrepancies. For down velocity, the filters diverged from each other. Regarding the Euler angles, presented in Figure 15, the filters showed similar results for all three axes.

#### 5.2.2. PINA-M

**Figure 16:**
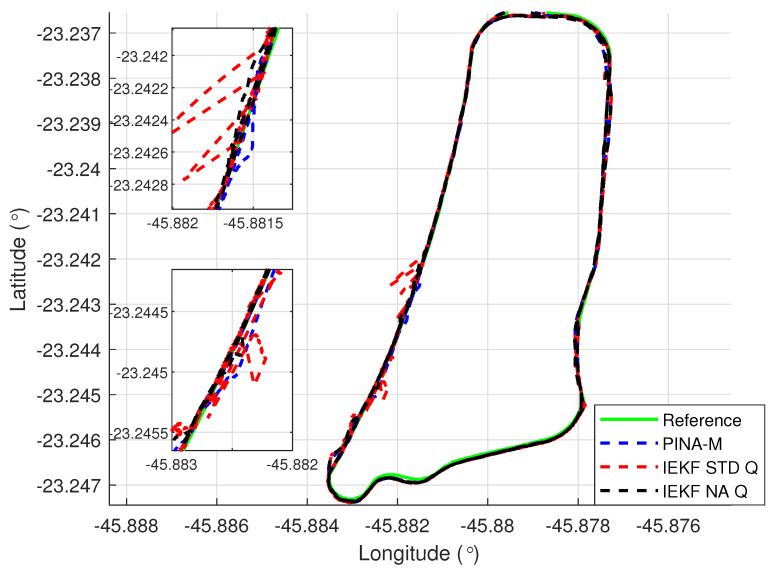
Geodetic position.

**Figure 17:**
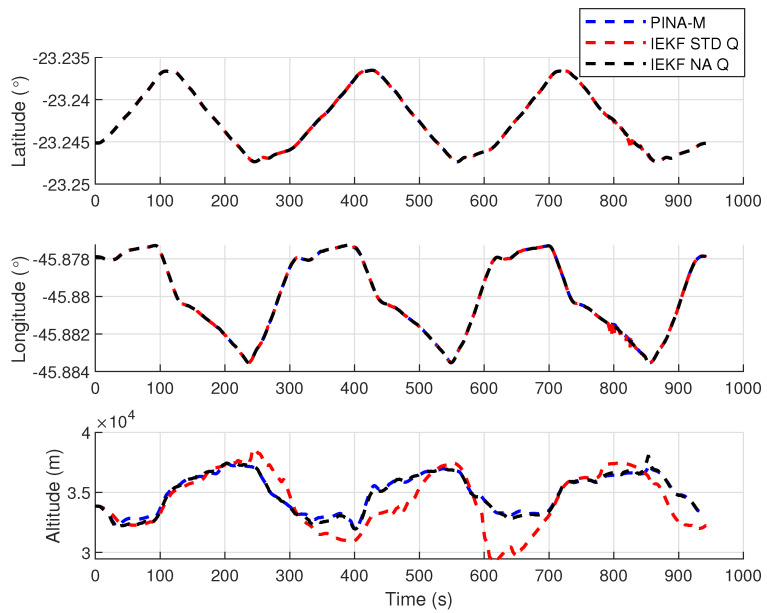
Positions in geodetic frame.

**Figure 18:**
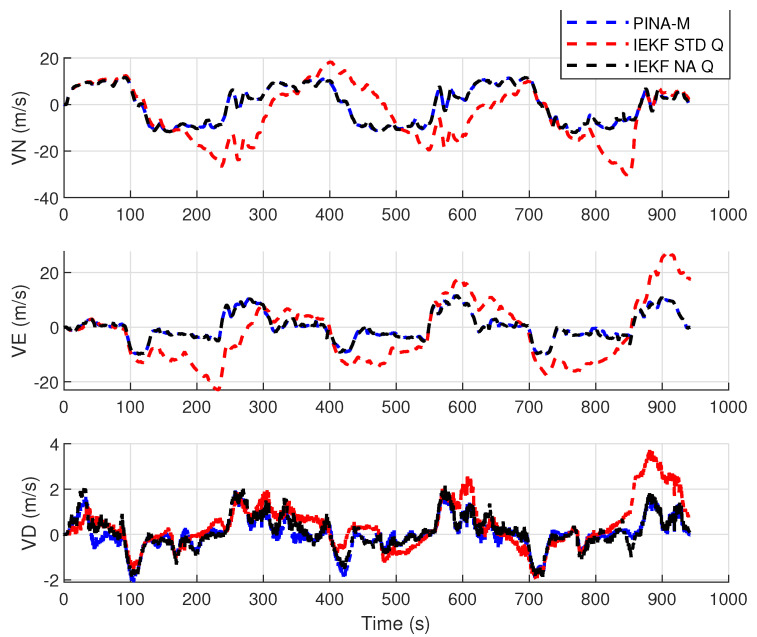
Velocities in navigation frame.

**Figure 19:**
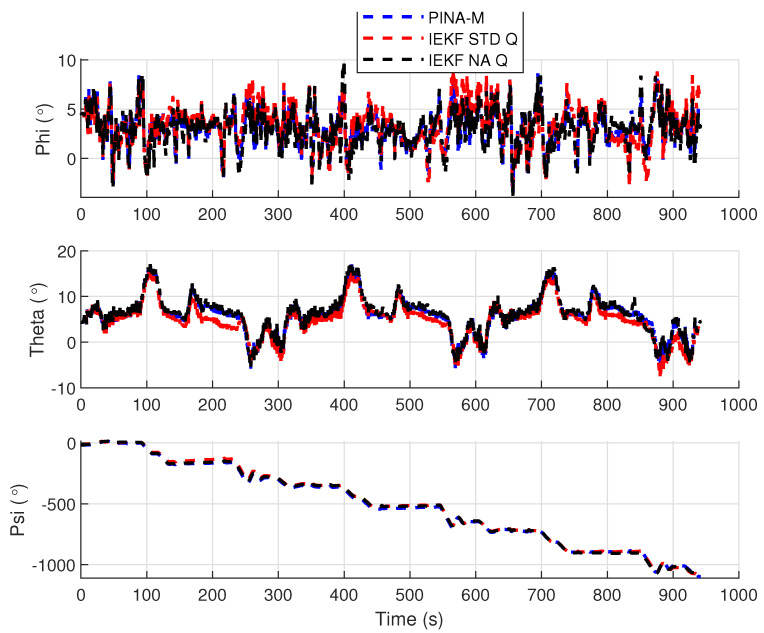
Euler angles.

Figure 16 presents the results of the INS/GNSS integration for the PINA-M in the geodetic reference frame. The NA tuning maintains the trajectory even during periods of GNSS signal interference, achieving better results compared to the platform itself, as highlighted in the enlarged areas. In Figure 17, the STD tuning shows degradation in altitude throughout the trajectory, as well as in latitude and longitude during GNSS outages, whereas the NA tuning remains closely aligned with the platform’s results. In Figure 18, the NA-tuned filter closely follows the platform’s results, while the STD tuning exhibits discrepancies across all three axes. For the Euler angles, presented in Figure 19, the STD tuning diverges from both the platform results and the NA tuning, particularly in the ϕ and θ axes.

## 6. Discussion

The results demonstrated that the proposed innovative tuning method achieved satisfactory results compared to commercial platforms, even when different sensor technologies were used. The method aims to quickly determine the appropriate values for the Q matrix based on sensor datasheet values, thus eliminating the need for an experienced designer, as well as any heuristic assumptions or initial guesses.

The datasheet information provided by manufacturers, especially for lower-cost sensors, often does not perfectly correspond to the actual performance of the sensors. This discrepancy necessitates the performance of new Allan variance tests to obtain values closer to real-world performance. These tests are time-consuming, require a controlled environment, and demand expertise from the person conducting them. The proposed method converts the datasheet values into more accurate real-world values, streamlining the process and removing the need for a specialist.

Additionally, the implementation of the method on platforms with different sensor technologies, such as FOGs and MEMS, highlights the robustness and flexibility of the approach. Even in scenarios where the sensors have significantly different noise characteristics, the proposed tuning method successfully maintained trajectory accuracy, particularly during periods of GNSS signal degradation.

Future work will focus on further optimizing the tuning parameters. Specifically, the Q matrix will be made adaptive to temperature variations, as inertial sensors are particularly sensitive to these changes. This adjustment would allow for even more precise tuning under variable operational conditions, potentially further improving the performance of inertial navigation systems.

Moreover, incorporating Real-Time Kinematic (RTK) data in future experiments could provide an even higher level of accuracy and serve as a more precise reference for evaluating the proposed method. A direct comparison of the proposed method with other recent automatic Kalman filter tuning methods, including those that use machine learning or genetic algorithms, would also be beneficial.

## 7. Conclusions

This work presents an analysis of the tuning methods for Extended Kalman Filters (EKFs) used in INS/GNSS integration. By comparing the standard (STD) tuning approach with a newly proposed approach (NA), the study demonstrates the efficacy of the NA tuning in maintaining trajectory accuracy, especially during periods of GNSS signal degradation. The NA tuning consistently shows better alignment with the platform results across various metrics, including altitude, latitude, longitude, velocities, and Euler angles. The findings indicate that the proposed tuning method not only enhances the performance of inertial navigation systems by providing more reliable and precise navigation solutions but also offers significant improvements in tuning efficiency. The proposed method achieves appropriate values for the Q matrix based on the datasheet specifications of the inertial sensors, streamlining the tuning process and reducing the time required to reach these desired values.

## Figures and Tables

**Table 1:** Accelerometer specifications.

Platform	In-Run Bias Stability	Noise Density	Bandwidth (−3 dB)	Sample Rate
PINA-F	<1 μg	<70 μg/$\sqrt{\mathrm{Hz}}$	39 Hz	100 Hz
PINA-M	13 μg	10 μg/$\sqrt{\mathrm{Hz}}$	80 Hz	200 Hz

**Table 2:** Gyroscope specifications.

Platform	In-Run Bias Stability	Noise Density	Bandwidth (−3 dB)	Sample Rate
PINA-F	1°/h	0.0004°/s/$\sqrt{\mathrm{Hz}}$	32 Hz	100 Hz
PINA-M	2.5°/h	0.003°/s/$\sqrt{\mathrm{Hz}}$	80 Hz	200 Hz

**Table 3:** Variances for DEKF.

		PINA-F		PINA-M	
Parameter	Axis	STD	NA	STD	NA
Position	*x*	0	$6.1\times 10^{-10}$	0	$3.47\times 10^{-10}$
	*y*	0	$2.0\times 10^{-10}$	0	$1.13\times 10^{-10}$
	*z*	0	$1.5502\times 10^{4}$	0	$8.80\times 10^{4}$
Velocity	*x*	$4.7155\times 10^{-5}$	$1.4308\times 10^{-4}$	$1.9247\times 10^{-6}$	$8.1222\times 10^{-5}$
	*y*	$4.7155\times 10^{-5}$	$4.2824\times 10^{-5}$	$1.9247\times 10^{-6}$	$2.4310\times 10^{-5}$
	*z*	$4.7155\times 10^{-5}$	$1.7016\times 10^{-4}$	$1.9247\times 10^{-6}$	$9.6598\times 10^{-5}$
Attitude	*x*	$4.8738\times 10^{-9}$	$3.6\times 10^{-10}$	$5.4831\times 10^{-7}$	$2.05\times 10^{-10}$
	*y*	$4.8738\times 10^{-9}$	$4.2\times 10^{-10}$	$5.4831\times 10^{-7}$	$2.38\times 10^{-10}$
	*z*	$4.8738\times 10^{-9}$	$3.0\times 10^{-11}$	$5.4831\times 10^{-7}$	$1.70\times 10^{-11}$

**Table 4:** Variances for IEKF.

		PINA-F		PINA-M	
Parameter	Axis	STD	NA	STD	NA
Accel Errors	*x*	$4.7155\times 10^{-5}$	$6.5378\times 10^{-5}$	$1.9247\times 10^{-6}$	$3.3675\times 10^{-4}$
	*y*	$4.7155\times 10^{-5}$	$6.5378\times 10^{-5}$	$1.9247\times 10^{-6}$	$3.3675\times 10^{-4}$
	*z*	$4.7155\times 10^{-5}$	$6.5378\times 10^{-5}$	$1.9247\times 10^{-6}$	$3.3675\times 10^{-4}$
Gyro Errors	*x*	$4.8738\times 10^{-9}$	$5.0478\times 10^{-6}$	$5.4831\times 10^{-7}$	$1.1584\times 10^{-5}$
	*y*	$4.8738\times 10^{-9}$	$5.0478\times 10^{-6}$	$5.4831\times 10^{-7}$	$1.1584\times 10^{-5}$
	*z*	$4.8738\times 10^{-9}$	$5.0478\times 10^{-6}$	$5.4831\times 10^{-7}$	$1.1584\times 10^{-5}$

## Data Availability

The original contributions presented in this study are included in this article.

## References

[1] Groves P.D. (2013). Principles of GNSS, Inertial, and Multisensor Integrated Navigation Systems.

[2] Simon D. (2006). Optimal State Estimation: Kalman, H Infinity, and Nonlinear Approaches.

[3] Zhang A., Atia M.M. (2020). An efficient tuning framework for Kalman filter parameter optimization using design of experiments and genetic algorithms. Navig. J. Inst. Navig..

[4] Wondosen A., Debele Y., Kim S.K., Shi H.Y., Endale B., Kang B.S. (2023). Bayesian optimization for fine-tuning EKF parameters in UAV attitude and heading reference system estimation. Aerospace.

[5] Nasiri M., Birjand I., Zahiri S.H., Havangi R. (2016). Design an Adaptive Kalman Filter for INS/GPS based navigation for a vehicular system. Int. J. Comput. Sci. Inf. Secur..

[6] Mahmoud M., Alaa I., Wassal A., Noureldin A., Eldieb A. Tuning of the error covariance parameters in EKF-based INS/GPS systems: A practical approach. Proceedings of the International Conference on Mobile Mapping Technology (MMT).

[7] Goodall C., El-Sheimy N. (2007). Intelligent tuning of a Kalman filter using low-cost MEMS inertial sensors. Proceedings of the 5th International Symposium on Mobile Mapping Technology (MMT’07).

[8] Han S., Wang J., Knight N. (2009). Using allan variance to determine the calibration model of inertial sensors for GPS/INS integration. Proceedings of the 6th International Symposium on Mobile Mapping Technology.

[9] Grewal M.S., Andrews A.P. (2014). Kalman Filtering: Theory and Practice with MATLAB.

[10] Farrell J.A. (2008). Aided Navigation: GPS with High Rate Sensors.

[11] Mehra R. (1970). On the identification of variances and adaptive Kalman filtering. IEEE Trans. Autom. Control.

[12] Mehra R. (1972). Approaches to adaptive filtering. IEEE Trans. Autom. Control.

[13] Carew B., Belanger P. (1973). Identification of optimum filter steady-state gain for systems with unknown noise covariances. IEEE Trans. Autom. Control.

[14] Belanger P.R. (1974). Estimation of noise covariance matrices for a linear time-varying stochastic process. Automatica.

[15] Rajamani M.R. (2007). Data-Based Techniques to Improve State Estimation in Model Predictive Control. Ph.D. Thesis.

[16] Åkesson B.M., Jørgensen J.B., Poulsen N.K., Jørgensen S.B. (2008). A generalized autocovariance least-squares method for Kalman filter tuning. J. Process Control.

[17] Matisko P., Havlena V. (2013). Noise covariance estimation for Kalman filter tuning using Bayesian approach and Monte Carlo. Int. J. Adapt. Control. Signal Process..

[18] El-Sheimy N., Hou H., Niu X. (2007). Analysis and modeling of inertial sensors using Allan variance. IEEE Trans. Instrum. Meas..

[19] Wang D., Dong Y., Li Q., Li Z., Wu J. (2018). Using Allan variance to improve stochastic modeling for accurate GNSS/INS integrated navigation. GPS Solut..

[20] Shahrawy A., Radi A., Zahran S. (2024). INS/GPS KF Integration Performance Improvement Based on Accurate Inertial Sensors Stochastic Error Modelling. Przegląd Elektrotechniczny.

[21] Korniyenko O.V., Sharawi M.S., Aloi D.N. (2005). Neural network based approach for tuning Kalman filter. Proceedings of the 2005 IEEE International Conference on Electro Information Technology.

[22] Baek S., Liu C., Watta P., Murphey Y.L. (2017). Accurate vehicle position estimation using a Kalman filter and neural network-based approach. Proceedings of the 2017 IEEE Symposium Series on Computational Intelligence (SSCI).

[23] Li J., Hou J., Ning Y., Xing J. (2023). Research on CNN Fusion Processing Technology for GNSS/INS Special Area Navigation Application. Proceedings of the 2023 3rd International Conference on Electronic Information Engineering and Computer Communication (EIECC).

[24] Oshman Y., Shaviv I. Optimal tuning of a Kalman filter using genetic algorithms. Proceedings of the AIAA Guidance, Navigation, and Control Conference and Exhibit.

[25] Naseri F., Setoodeh P., Schaltz E. (2024). Online Tuning of Extended Kalman Filter Using Reinforcement Learning for Improved Battery State-of-Charge Estimation. Proceedings of the 2024 IEEE International Conference on Industrial Technology (ICIT).

[26] Robert C.P., Casella G., Casella G. (1999). Monte Carlo Statistical Methods.

[27] Kroese D.P., Taimre T., Botev Z.I. (2013). Handbook of Monte Carlo Methods.

[28] Feller W. (1991). An Introduction to Probability Theory and Its Applications, Volume 2.

[29] Durrett R. (2019). Probability: Theory and Examples.

[30] Savage P.G. (2000). Strapdown Analytics.

[31] Pham V.T., Nguyen V.T., Chu D.T., Tran D.T. (2015). 15-state extended Kalman filter design for INS/GPS navigation system. J. Autom. Control. Eng..

[32] Noureldin A., Karamat T.B., Georgy J. (2012). Fundamentals of Inertial Navigation, Satellite-Based Positioning and Their Integration.

[33] Kaplan E.D., Hegarty C.J. (2005). Understanding GPS: Principles and Applications.

[34] Hofmann-Wellenhof B., Lichtenegger H., Wasle E. (2012). GNSS—Global Navigation Satellite Systems: GPS, GLONASS, Galileo, and More.

[35] Leick A., Rapoport L., Tatarnikov D. (2015). GPS Satellite Surveying.

[36] Titterton D., Weston J.L. (2004). Strapdown Inertial Navigation Technology.

[37] Zhang F., Moore T., Harris C., Yu X. (2020). Multi-sensor navigation fusion: Inertial, optical flow, and GNSS. IEEE Trans. Aerosp. Electron. Syst..

[38] Gao Y., Groves P.D. (2017). Integrated GNSS/INS Systems. Inert. Navig. Integr..

[39] Qi H., Moore J.B. (2002). Direct Kalman filtering approach for GPS/INS integration. IEEE Trans. Aerosp. Electron. Syst..

[40] Falletti E., Rao M., Savasta S. (2011). The Kalman Filter and its Applications in GNSS and INS. Handbook of Position Location: Theory, Practice, and Advances.

[41] Christophersen H.B., Pickell R.W., Neidhoefer J.C., Koller A.A., Kannan S.K., Johnson E.N. (2006). A compact guidance, navigation, and control system for unmanned aerial vehicles. J. Aerosp. Comput. Inform. Commun..

[42] Ibrahim A., Abosekeen A., Azouz A., Noureldin A. (2023). Enhanced autonomous vehicle positioning using a loosely coupled INS/GNSS-based invariant-EKF integration. Sensors.

[43] Savage P.G. (2000). Strapdown Analytics.

[44] Efanov A.A., Ivliev S.A., Shagraev A.G. (2021). Welford’s algorithm for weighted statistics. Proceedings of the 2021 3rd International Youth Conference on Radio Electronics, Electrical and Power Engineering (REEPE).

[45] Nobach H. (2023). Practical Realization of Bessel’s Correction for a Bias-Free Estimation of the Auto-Covariance and the Cross-Covariance Functions. arXiv.

[46] Dobrushin R.L. (1956). Central limit theorem for nonstationary Markov chains. I. Theory Probab. Its Appl..

[47] Trevezas S., Limnios N. (2009). Variance estimation in the central limit theorem for Markov chains. J. Stat. Plan. Inference.

